# Nitrogen Mineralization of Selected Organic Materials and Their Combined Effects with Nitrogen Fertilizer on Spinach Yield

**DOI:** 10.3390/plants13141974

**Published:** 2024-07-19

**Authors:** Sibongiseni Mgolozeli, Adornis D. Nciizah, Isaiah I. C. Wakindiki, Fhatuwani N. Mudau

**Affiliations:** 1 Agricultural Research Council—Natural Resources and Engineering, Private Bag X79, Pretoria 0001, South Africa; nciizaha@arc.agric.za; 2 Department of Agriculture and Animal Health, University of South Africa, Roodepoort 1709, South Africa; iwakindiki@kcau.ac.ke (I.I.C.W.); mudauf@ukzn.ac.za (F.N.M.); 3 School of Graduate Studies, KCA University, Nairobi P.O. Box 56808-00200, Kenya; 4 School of Agricultural, Earth and Environmental Sciences, University of KwaZulu-Natal, Private Bag X01, Pietermaritzburg 3209, South Africa

**Keywords:** integrated nutrient management, soil texture, incubation study, glasshouse experiment, spinach yield

## Abstract

A 2-month incubation study was carried out using two soil types to determine the nitrogen mineralization of different inorganic–organic amendments. The following seven treatments (Ts) were established: T1 = control (no amendment), T2 = 5 g of dry algae per kg of soil (100%DA), T3 = 136 g of agri-mat per kg of soil (100%GAM), T4 = 61 g of ground grass per kg of soil (100%GG), T5 = 0.6 g of N using lime–ammonium nitrate (LAN) + 2.5 g of dry algae (50%DA50NF), T6 = 50%GAM50NF, and T7 = 50%GG50NF. Three samples per treatment were obtained at 0, 3, 7, 15, 30, 45, and 60 days for N mineral determination. A 2-month glasshouse experiment was established afterward with the following five treatments: T1 = control, T2 = 50%DA, T3 = 50%GAM, T4 = 50%GG, and T5 = 100 NF. The results indicate that nitrogen mineralization was significantly higher in organic–inorganic amendments compared with singular organic amendments. The percentage differences ranged from 157% to 195%. The 50%DA treatment increased the spinach yield by 20.6% in sandy loam and 36.5% in loam soil. It is difficult to fully recommend the 50%DA treatment without field-scale evaluation, but it is a promising option to be considered.

## 1. Introduction

Chemical fertilizers improve soil fertility and crop yields, but their excessive use can lead to global warming, a decline in soil organic matter and soil quality, eutrophication in estuaries, and the contamination of other water bodies via leaching and runoff [1,2,3]. Unlike chemical fertilizers, organic amendments may offer other benefits, including improved soil fertility by stimulating nutrient cycle mechanisms through the mineralization of nitrogen and phosphorus [4,5,6]. In addition, organic amendments can help in the formation and stabilization of soil aggregates, carbon sequestration, improving the microbial population and enzyme activity, and improving the functioning of the agroecosystem [6,7,8]. These benefits enable longer-term soil health and sustainable agricultural practices, which, in turn, support increased soil fertility and improved crop yields [6]. According to Diacono and Montemurro [9], soil fertility may be defined as the capacity of the soil to provide physical, chemical, and biological needs for the growth of plants for productivity, reproduction, and quality, which depends on the plant and soil type, land use, and climatic conditions. 

Nitrogen mineralization is a process that involves the transformation (organic matter breakdown by microorganisms) of organic nitrogen into inorganic forms (ammonium (NH_4_^+^) and nitrate (NO_3_^−^) ions) that are easily absorbed by plants and is, therefore, essential for soil fertility and plant growth [10,11]. The transformation or breakdown of organic nitrogen compounds such as amino acids and proteins into NH_4_^+^ is called ammonification, the first step of the N mineralization process. The second step is called nitrification, a process where NH_4_^+^ is oxidized to nitrite (NO_2_^−^) by Nitrosomonas bacteria and then further oxidized to NO_3_^−^ by Nitrobacter bacteria [10]. The quality and quantity of organic residues (especially their carbon-to-nitrogen ratio), retained or applied in the field as mulch [12], determines the N mineralization rate of any given soil [10,13]. Generally, the rate of nitrogen mineralization for any organic material is governed by many soil factors such as moisture [10,14,15], texture and structure [10], microbial activity [16,17], soil temperature [1,14], soil pH [10], the C-to-N ratio [10,17,18], the lignin-to-N ratio, and the polyphenols-to-N ratio [10,18].

The examination of the nitrogen (N) release capacity from organic materials is of utmost importance in the quantification of the N supply capacity and fertilizer values of organic amendments [8]. The use of algae biomass as a biofertilizer has been recommended as an efficient technique to increase the crop growth parameters and total yields of many plants due to its high nutrient content, especially N [19,20]. Algae biomass is a nutrient-dense soil conditioner that has the potential to increase the soil microbial population and enzyme activity, which elevates inorganic forms of nitrogen levels in the soil, such as ammonium and nitrate, through mineralization [21]. Mgolozeli et al. [22] reported that algae residues have the capacity to increase water absorption and water-holding capacity while improving the nutrient status and electrical conductivity of agri-mats, making them more beneficial not only as a runoff-combating technology but also as a soil conditioner. Agri-mats are novel and innovative mulching materials that can increase soil organic matter content [23] and improve crop biomass yields [24]. Agri-mats are solid organic mulch mats derived from agricultural waste and other organic waste materials, such as grass, weed biomass, municipal sewage sludge, algae residues, bagasse, and forestry waste, created by hot or cold pressing biomass into soil mats. These environmentally friendly mulches serve as effective ground cover while releasing nutrients into the soil. Agri-mats provide remarkable benefits, including conserving soil and moisture, regulating temperature, improving soil structure and microbial activity, enhancing fertility, and increasing crop productivity, especially in drier regions. They suppress weeds, reducing herbicide needs and costs. As biodegradable materials, agri-mats decompose over time into a valuable soil organic matter source without hazardous residues and can eliminate or significantly cut costs for weeding compared with compost. Their utilization recycles agricultural and forestry wastes, aligning with conservation agriculture and land rehabilitation principles [22,23,24].

Although the mineralization rates of algae and many other organic residues have been studied by several authors [4,8,19,20], research on the nitrogen mineralization rate of agri-mats is scanty. Moreover, in South Africa and the world at large, there is a paucity of information on the combined effects of agri-mats and inorganic nitrogen fertilizer on soil fertility and crop growth in different soil textural classes. 

Soil texture and the particle size of organic amendments have a direct influence on N mineralization, with finer organic particle sizes resulting in more rapid N release than larger or coarser particles [10,18]. Meanwhile, N mineralization rates are often lower in fine-textured (clay) soils compared with coarse-textured soils [25,26]. Generally, soil particle size (texture) has a major influence on soil carbon and nitrogen dynamics [27]. The practice of applying organic amendments, such as grass, algal biomass, crop and/or forestry residues, etc., to soil could play a significant role in improving not only soil fertility but overall soil health and crop productivity [5,10,18,19,28]. According to Shahid and Al-Shankiti [19], organic amendments increase the efficiency of inorganic fertilizers via positive interactions with soil biological, chemical, and physical properties. The objectives of the current study were (i) to investigate the nitrogen mineralization rate of selected organic residues (ground agri-mats, dry micro-algal residues, and grass mulch) and their combined effects with lime–ammonium nitrate, and (ii) to investigate the combined effect of selected organic residues with lime–ammonium nitrate on the growth and biomass yield of spinach (*Spinacia oleracea* L.) in loamy sand and loam soils. Spinach was chosen as a test crop because it is a staple food in South Africa, along with maize, and is more suitable for pot experiments than maize.

## 2. Results

### 2.1. Incubation Study

#### 2.1.1. Sandy Loam Soil from Pretoria

**Figure 1:**
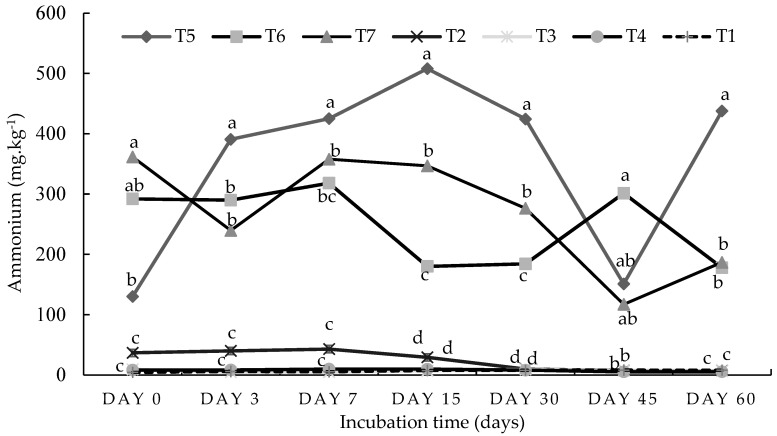
Ammonium dynamics in a sandy loam soil from Pretoria, Gauteng. Means with the same letter on the same day indicate there were no significant (*p* < 0.05) differences between the treatment means. The statistical differences were detected using the LSD test at a 0.05 confidence level; *n* = 42. LSD_DAY0_ = 0.0347, LSD_DAY3_ = 0.00629, LSD_DAY7_ = 0.0445, LSD_DAY15_ = 0.0076, LSD_DAY30_ = 0025, LSD_DAY45_ = 0.0087, and LSD_DAY60_ = 0.0061. T1 = control (no amendment), T2 = 5 g of dry algae per kg of soil (100%DA), T3 = 136 g of agri-mat per kg of soil (100%GAM), T4 = 61 g of ground grass per kg of soil (100%GG), T5 = 0.6 g of N using lime–ammonium nitrate (LAN) + 2.5 g of dry algae (50%DA50NF), T6 = 50%GAM50NF, and T7 = 50%GG50NF.

The results from the current incubation study indicate that the ammonium content was significantly (*p* < 0.05) higher in the combined treatments (half-organic and half-inorganic fertilizers) than in the 100% organic amendment treatments throughout the incubation period (Figure 1). The combined 50%DA50NF treatment resulted in a significantly (*p* < 0.05) higher level of nitrogen mineralization (507.9 mg N kg^−1^) on day 15. The initial value of 130.08 mg kg^−1^ recorded under the same treatment was significantly (*p* < 0.05) lower compared with the other treatment combinations. The amount of ammonium, which was released from all organic amendment treatments without a nitrogen fertilizer, remained constant throughout the incubation study, except for in the 100% dry algae treatment (Figure 1). Therein, the mineralization rate decreased on day 7 and continued to drop until day 30. The lowest significant (*p* < 0.05) value of ammonium was recorded under the control treatment on all sampling days. 

**Figure 2:**
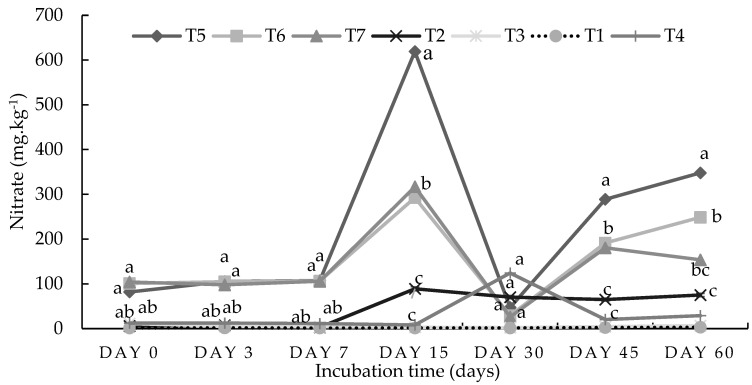
Nitrate dynamics in sandy loam soil from Pretoria, Gauteng. Means with the same letter on the same day indicate there were no significant (*p* < 0.05) differences between the treatment means. The statistical differences were detected using the LSD test at a 0.05 confidence level; *n* = 42. LSD_DAY0_ = 0.0058, LSD_DAY3_ = 0.0095, LSD_DAY7_ = 0.0054, LSD_DAY15_ = 0.0343, LSD_DAY30_ = 6847, LSD_DAY45_ = 0.0753, and LSD_DAY60_ = 0.0634. T1 = control (no amendment), T2 = 5 g of dry algae per kg of soil (100%DA), T3 = 136 g of agri-mat per kg of soil (100%GAM), T4 = 61 g of ground grass per kg of soil (100%GG), T5 = 0.6 g of N using lime–ammonium nitrate (LAN) + 2.5 g of dry algae (50%DA50NF), T6 = 50%GAM50NF, and T7 = 50%GG50NF.

Similar to ammonium, the highest significant (*p* < 0.05) amount of nitrate (619 mg kg^−1^) was recorded under the 50% dry algae (DA) and 50% nitrogen fertilizer treatments on day 15 (Figure 2). For the first seven days, all three combined organic–inorganic treatments (50%DA50NF; 50%GAM50NF; and 50%GG50NF) remained constant, with an average value of 100 mg kg^−1^ (Figure 2). Similarly, the other three organic amendments without an inorganic fertilizer remained constant, with an average value of 11 mg kg^−1^ for the first seven days. The dynamics of nitrate remained the same for all treatments from day seven until the final day of the incubation. Generally, the lowest significant (*p* < 0.05) nitrate values were recorded in the control treatment, while the highest values were recorded in the 50%DA50NF treatment combination (Figure 2).

#### 2.1.2. Loam Soil from Pretoria

**Figure 3:**
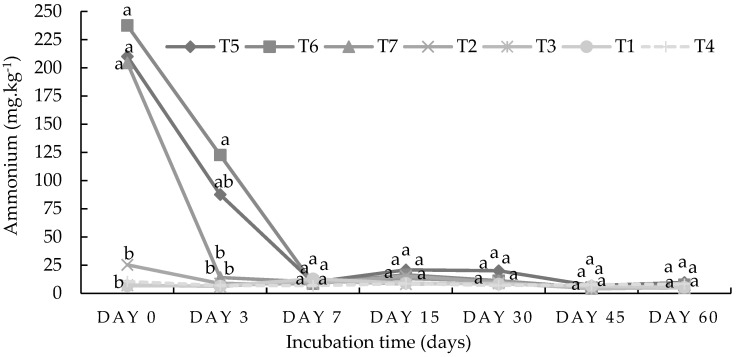
Ammonium dynamics in a loam soil from Durban, Kwa-Zulu Natal. Means with the same letter on the same day indicate there were no significant (*p* < 0.05) differences between the treatment means. The statistical differences were detected using the LSD test at a 0.05 confidence level; *n* = 42. LSD_DAY0_ = 0.0089, LSD_DAY3_ = 0.0265, LSD_DAY7_ = 0.8021, LSD_DAY15_ = 0.7432, LSD_DAY30_ = 5405, LSD_DAY45_ = 0.5798, and LSD_DAY60_ = 0.6453. T1 = control (no amendment), T2 = 5 g of dry algae per kg of soil (100%DA), T3 = 136 g of agri-mat per kg of soil (100%GAM), T4 = 61 g of ground grass per kg of soil (100%GG), T5 = 0.6 g of N using lime–ammonium nitrate (LAN) + 2.5 g of dry algae (50%DA50NF), T6 = 50%GAM50NF, and T7 = 50%GG50NF.

The results show that the organic amendment had a significant (*p* < 0.05) influence on nitrogen mineralization in loam soil during the first three days of the incubation study (Figure 3). The highest significant (*p* < 0.05) value of ammonium 237.53 mg kg^−1^ was recorded under the 50%DA treatment combination on day 0 but dropped sharply after 2 days to 122.65 mg kg^−1^ and further dropped to about 9.88 mg kg^−1^. The combined organic and inorganic fertilizer treatments produced significantly (*p* < 0.05) higher ammonium values than the singular organic fertilizer treatments on day 0 and day 3. From day 7 until day 60, there were no significant (*p* < 0.05) differences between any of the treatments including the control (Figure 3).

**Figure 4:**
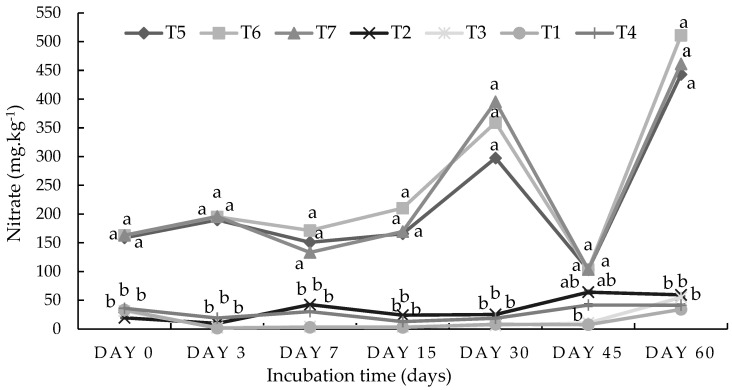
Nitrate dynamics in a loam soil from Durban, Kwa-Zulu Natal. Means with the same letter on the same day indicate there were no significant (*p* < 0.05) differences between the treatment means. The statistical differences were detected using the LSD test at a 0.05 confidence level; *n* = 42. LSD_DAY0_ = 0.0139, LSD_DAY3_ = 0.0201, LSD_DAY7_ = 0.0098, LSD_DAY15_ = 0.0071, LSD_DAY30_ = 0045, LSD_DAY45_ = 0.3452, and LSD_DAY60_ = 0.0067. T1 = control (no amendment), T2 = 5 g of dry algae per kg of soil (100%DA), T3 = 136 g of agri-mat per kg of soil (100%GAM), T4 = 61 g of ground grass per kg of soil (100%GG), T5 = 0.6 g of N using lime–ammonium nitrate (LAN) + 2.5 g of dry algae (50%DA50NF), T6 = 50%GAM50NF, and T7 = 50%GG50NF.

Similar to ammonium, the results show that the nitrate content was significantly (*p* < 0.05) higher in the combined treatments (half-organic and half-inorganic fertilizers) than in the 100% organic amendments treatments throughout the incubation period (Figure 4). The highest significant (*p* < 0.05) value of nitrate (511 mg kg^−1^) was recorded under the 50%DA50NF treatment combination on the final day of the incubation study. Conversely, the lowest significant (*p* < 0.05) nitrate value of 41.4 mg kg^−1^ on the last day was recorded under the control treatment. Significant nitrate addition to the soil due to the mineralization process was only observed in the combined organic amendment treatments (Figure 4).

The results from the incubation study show that at the peak of nitrogen mineralization (day 15 in Figure 1), the amount of ammonium ions released under the 50%DA50NF treatment in the sandy loam soil was 178% higher compared with the 100%DA treatment. For the 50%GG50NF and 50%GAM50NF treatment combinations, the percentage differences in ammonium ions were 192% and 184%, respectively. Meanwhile, the amount of nitrate ions released at the peak (day 15 in Figure 2) under the 50%DA50NF treatment was 159% higher compared with the 100%DA treatment. For the 50%GG50NF and 50%GAM50NF treatment combinations, the percentage differences in the nitrate amount were 175% and 189%, respectively, compared with their singular organic amendments’ counterparts. In the loam soil, the peak of nitrogen mineralization occurred on day 0 (Figure 3) for ammonium and day 60 for nitrate (Figure 4). The amount of ammonium that was released under the 50%DA50NF treatment was 186% higher compared with the 100%DA treatment. Meanwhile, for the 50%GG50NF and 50%GAM50NF treatment combinations, the differences in nitrate ions were 184% and 157%, respectively, compared with their singular organic amendments’ counterparts. The amount of nitrate that was released at the peak under the 50%DA50NF treatment was 161% higher compared with the 100%DA treatment. For the 50%GG50NF and 50%GAM50NF treatment combinations, the percentage differences in nitrate ions were 167% and 153%, respectively. 

### 2.2. Glasshouse Experiment

**Figure 5:**
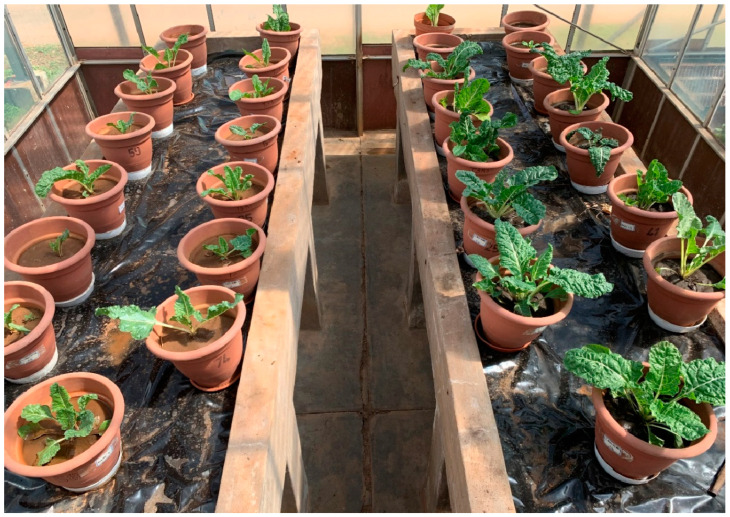
Effects of various integrated nutrient management strategies on spinach growth planted in loam and sandy loam soils.

**Figure 6:**
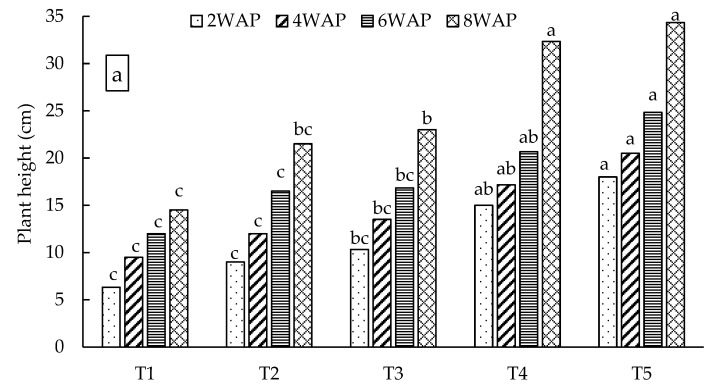

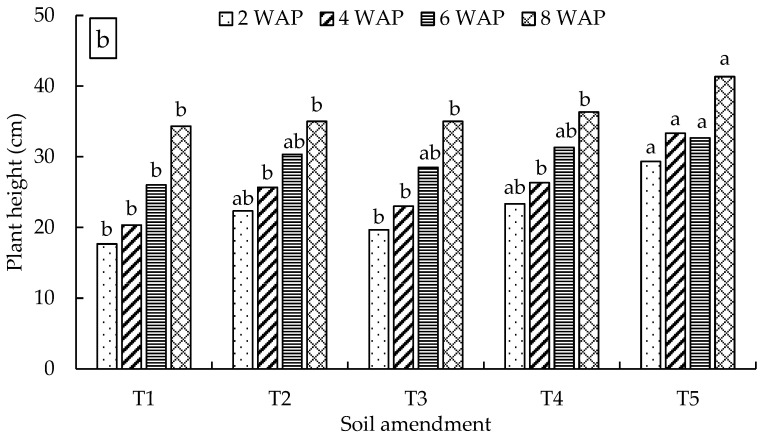
Effects of organic–inorganic amendments in sandy loam (**a**) and loam (**b**) soils on spinach height. WAP = weeks after planting. Means with the same letter within the same measuring period are not significantly (*p* < 0.05) different from each other. The statistical differences were detected using the LSD test at a 0.05 confidence level; *n* = 30. LSD_2WAP(a)_ = 0.0065, LSD_4WAP(a)_ = 0.0049, LSD_6WAP(a)_ = 0.0031, LSD_8WAP(a)_ = 0.0087, LSD_2WAP(b)_ = 0.0043, LSD_4WAP(b)_ = 0.0019, LSD_6WAP(b)_ = 0.0086, and LSD_8WAP(b)_ = 0.0081. T1 = control (no amendment), T2 = 50%GG50NF, T3 = 50%GAM50NF, T4 = 100NF, and T5 = 50%DA50NF.

The combination of organic–inorganic amendments and singular application of inorganic fertilizer had a significant (*p* < 0.05) effect on all measured spinach crop growth parameters in both soil types. Figure 5 shows the spinach growth during the third week after planting, with a higher biomass on the right (plants in loam soil) and a lower biomass on the left (in sandy loam soil). The lowest plant height values were recorded during the second week after planting (2WAP) in both soil types (6.3 cm for sandy loam soil and 17.7 cm for loam soil) under the control treatment (Figure 6). Conversely, the tallest plants were recorded under the combined treatment of nitrogen fertilizer and dry algae (50%DA50NF) during the eighth week after planting (8WAP), with plant height values of 34.3 cm and 41.3 cm in sandy loam and loam soils, respectively. In sandy loam soil (Figure 6a), the 50%DA50NF treatment consistently produced significantly (*p* < 0.05) taller plants compared with the other treatments at all measuring times, except during the eighth week after planting (8WAP). The difference between the 50%DA50NF and 150 kg N ha^−1^ (100NF) treatments at 8WAP was insignificant, with the former producing 2 cm taller plants compared with the latter (Figure 6a). The control treatment produced significantly (*p* < 0.05) lower plant height values in both sandy loam and loam soils at all four (2, 4, 6, and 8 WAP) measuring times (Figure 6).

**Figure 7:**
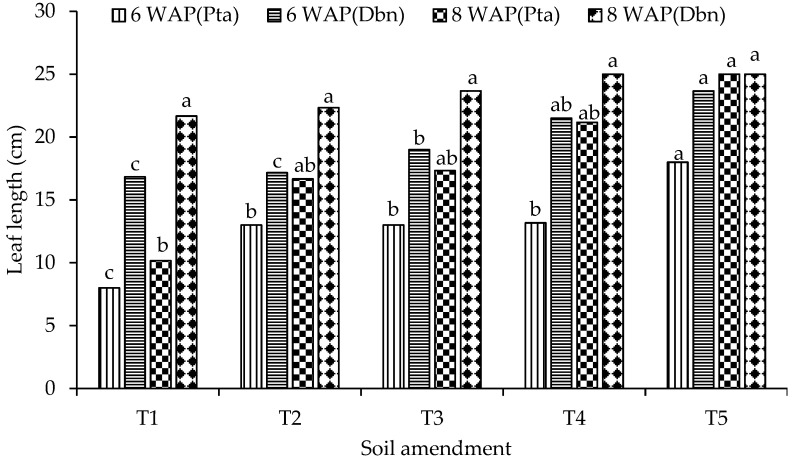
Effects of organic–inorganic amendments on spinach leaf length planted in sandy loam and loam soils. WAP = weeks after planting, Pta = Pretoria sandy loam soil, and Dbn = Durban loam soil. Means with the same letter within the same measuring time are not significantly (*p* < 0.05) different from each other. The statistical differences were detected using the LSD test at a 0.05 confidence level; *n* = 30. LSD_6WAP(Pta)_ = 0.0067, LSD_6WAP(Dbn)_ = 0.0008, LSD_8WAP(Pta)_ = 0.0032, and LSD_8WAP(Dbn)_ = 0.5466. T1 = control (no amendment), T2 = 50%GG50NF, T3 = 50%GAM50NF, T4 = 100NF, and T5 = 50%DA50NF.

Organic–inorganic amendments and inorganic fertilizer alone had a more pronounced effect on spinach growth in the last month (6WAP and 8WAP) in both soil types. Spinach leaves were significantly (*p* < 0.05) longer in pots with loam soil than in pots with sandy loam soil (Figure 7). In sandy loam soil, the control treatment produced significantly (*p* < 0.05) lower leaf length values at 6WAP (8 cm) and 8WAP (10.2 cm) compared with all other treatments. The highest significant values of spinach leaf length were observed under the 50%DA50NF treatment at 6WAP (18 cm) and 8WAP (25 cm). There were no significant differences between the 50%GG50NF (13 cm), 50%GAM50NF (13 cm), and 100NF (13.2 cm) treatments at 6WAP (Figure 7).

**Figure 8:**
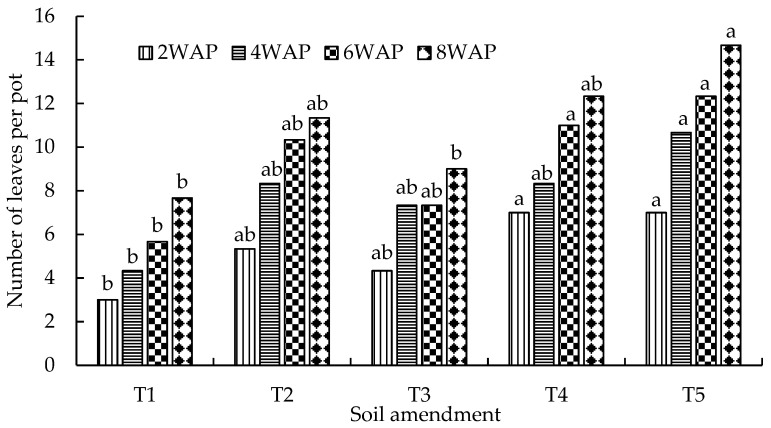
Effects of organic–inorganic amendments on the number of leaves of spinach planted in sandy loam soil. WAP = weeks after planting. Means with the same letter within the same measuring time are not significantly (*p* < 0.05) different from each other. The statistical differences were detected using the LSD test at a 0.05 confidence level; *n* = 30. LSD_2WAP_ = 0.0053, LSD_4WAP_ = 0.0091, LSD_6WAP_ = 0.0077, and LSD_8WAP_ = 0.0036. T1 = control (no amendment), T2 = 50%GG50NF, T3 = 50%GAM50NF, T4 = 100 NF, and T5 = 50%DA50NF.

The differences between the three treatments were, again, not significant at 8WAP. The leaf length values for the 50%GG50NF, 50%AG50NF, and 100NF treatments were 16.7 cm, 17.3 cm, and 21.2 cm, respectively. Similar to leaf length, the number of spinach leaves in the sandy loam soil was significantly (*p* < 0.05) influenced by the organic–inorganic amendments (Figure 8). Although the number of leaves was generally higher in the 50%GG50NF and 50%GA50NF treatments than in the control, the differences were not statistically significant between the two treatment combinations at 2, 4, and 6WAP. The number of leaves recorded in the 50%DA50NF treatment was 50% higher than the number of leaves recorded in the control treatment (Figure 8).

**Figure 9:**
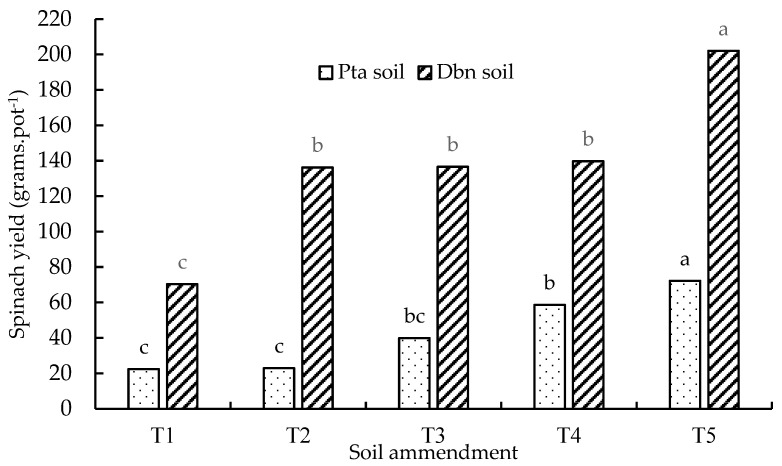
Yield biomass of spinach planted in loamy sand (Pretoria) and loam (Durban) soils treated with different soil amendments. Treatment means with the same letter for the soil type are not significantly (*p* < 0.05) different from each other. The statistical differences were detected using the LSD test at a 0.05 confidence level; *n* = 30. LSD_Pta_ = 0.0002; LSD_Dbn_ = 0.0045. T1 = control (no amendment), T2 = 50%GAM50NF, T3 = 50%GG50NF, T4 = 100 NF, and T5 = 50%DA50NF.

Similar to all the measured plant growth parameters, the inorganic–organic amendments and singular compound fertilizer had a significant (*p* < 0.05) effect on the spinach biomass yield in both loam soil and sandy loam soil (Figure 9). The spinach biomass yield was generally higher in pots filled with loam soil than in pots with sandy loam soil (Figure 9). Both soil types had the lowest significant (*p* < 0.05) values under the control treatment, at 22.4 g per pot for sandy loam soil and 70.3 g per pot for loam soil. The spinach yield in loam soil was more than three times higher than in sandy loam soil under the control treatment. The highest performing (50%DA50NF) treatment produced significantly (*p* < 0.05) higher yields in both soil types, i.e., 72.2 g per pot for sandy loam soil and 202.1 g per pot for loam soil. The biomass yield of spinach was 129.9 g per pot higher in loam soil than in sandy loam soil. No significant differences were observed between the 50%GAM50NF (136.2 g per pot), 50%GG50NF (136.6 g per pot), and 100NF (139.7 g per pot) treatments in loam soil. However, there were significant (*p* < 0.05) differences between the 50%GAM50NF (22.9 g per pot) and 100NF (58.7 g per pot) treatments in sandy loam soil (Figure 9). The results of the current study suggest that the 50%DA50NF treatment was the best-performing treatment for producing a higher biomass yield since it consistently outperformed the other treatments, even in terms of plant height, leaf length, and number of leaves, regardless of the soil type.

## 3. Discussion

### 3.1. Nitrogen Mineralization of Organic–Inorganic Amendments

The current incubation study aimed to investigate the nitrogen mineralization of two contrasting soil types using different organic-inorganic amendments over a period of two months (Table 1 and Table 2). Although the loam soil was more fertile compared with the sandy loam soil (Table 3), the incubation study results show that the nitrogen (N) mineralization rate (both ammonium and nitrate) was higher in sandy loam soil (Figure 1, Figure 2, Figure 3 and Figure 4). The higher mineralization rate in sandy loam soil is mostly ascribed to the differences observed in the clay content (9.3% vs. 24.3%) and least to the differences in the C–N ratio (9.22 vs. 11.50) compared with the loam soil (Table 3). The results of the current study are in line with the findings of Hassink et al. [26] and Bechtold and Naiman [25], who reported that N mineralization rates are often lower in fine-textured (clay) soils compared with coarse-textured soils. Moreover, according to Qiu et al. [29], the organic matter in clay soil is more resistant to decomposition than that in sandy soil. The higher clay content in loam soil has many micropores, which may limit the access and movement of microorganisms to organic residues for nitrogen mineralization [2,26,27,29]. Singh et al. [30] assessed three different soil clay fractions (SCFs) in the decomposition of wheat residues at 22 °C over a 36-day incubation period. The authors found that allophanic SCFs had the least microbial activity due to a higher proportion of micropores (75%).

The C–N ratio of both soil types was generally considered low since it was below 20 [31]. Soils with a low C–N ratio could accelerate the process of the microbial decomposition of organic matter and nitrogen mineralization, which may not be conducive to carbon sequestration [32]. Meanwhile, combining organic residues with a low C–N ratio and those with a high C–N ratio could create a conducive environment for carbon sequestration [6,9,32]. Bengtsson et al. [17] reiterated that soils with a high C–N ratio are characterized by the rapid immobilization of nitrogen, while soils with a low C–N ratio are characterized by slower immobilization and a surplus of available ammonium. The gradual decline in the ammonium mineralization observed between day 0 and day 7 in the loam soil (Figure 3) could be attributed to nitrogen immobilization [33]. Immobilization is the opposite of mineralization, and the difference between the two is referred to as net mineralization [2,33]. Apart from the clay percentage and C–N ratio, there were various factors that could have contributed to the differences in mineral N dynamics between the two soil types and overall fertility status. According to Malobane [33], soil fertility is a complex term that is influenced by a combination of factors such as soil texture, soil reaction, soil depth, nutrient content, soil microbial activity, organic matter content and composition, and the availability of potentially toxic substances.

The biochemical composition and physical structure of organic residues may also influence overall soil fertility and crop productivity [34]. The C–N ratio of organic amendments plays a significant role in the mineralization process, as observed in the current study [10,18]. Dried algae had a significantly (*p* < 0.05) higher mineralization rate, possibly due to its lower C–N ratio (6.6), as seen in Table 2, compared with ground agri-mat (184.0) and ground grass (75.7). These findings concur with the results obtained by Masunga et al. [35], who reported that soil treated with fresh white clover had a significantly higher mineralization rate due to its significantly low C–N ratio of 7.4 compared with other organic amendments they studied, such as polar tree compost, vegetable, fruit, and yard manure waste, and fresh dairy cattle manure, which had C–N ratios of 11.4, 9.1, and 12.3, respectively. A low C–N ratio is an indicator of high-quality organic matter, which supports and sustains microorganisms for N mineralization and other microbial activities that promote plant growth [35]. Organic amendments with high N contents and low C–N ratios, like algae and farmyard manure, can mineralize sufficient N to satisfy the growth demands of plants [6]. 

Among the three selected organic amendments (dried algae, ground agri-mat, and ground grass), ground agri-mat had the lowest nitrogen mineralization rate (Figure 1 and Figure 2), possibly due to its higher C–N ratio (Table 2). The low N mineralization of agri-mats could also be attributed to their high lignin content since they are manufactured from forestry residues (18, 24]. The agri-mat was used as a soil amendment, mixed in with the soil, while its practical use is to be applied as a mulch. The high C–N ratio of agri-mat (184) results in immobilization when mixed into soil, but when applied as a mulch, this effect is less pronounced. However, Mgolozeli et al. [22] concluded that the incorporation of algae into agri-mat technology could improve the functionality and value of agri-mats for agricultural purposes. The findings of the current study (Figure 1 and Figure 2) validate this suggestion, mainly due to the high nutrient content and low C–N ratio of algae residues (Table 2). The combination of organic amendments with different C–N ratios can help synchronize N mineralization with plant N demands [6]. Moreover, the incorporation of both algae and guinea grass during the agri-mat fabrication process may improve the C–N ratio of agri-mat, including nitrogen, phosphorus content, and many other essential elements for plant growth. The ground grass was generally the second-performing organic amendment, which had significantly higher ammonium and nitrate content on most days when measurements were performed (Figure 1 and Figure 4). Table 2 shows that ground grass had double the amount of nitrogen and phosphorus compared with ground agri-mat. Moreover, ground grass had a higher content of aluminum and iron than dried algae (Table 2). In both soil types, when organic and inorganic fertilizers were applied together, the mineralization rate was significantly higher compared with the treatments where organic amendments were singularly applied. This was observed in similar studies conducted by Abbasi and Khaliq [8] and Ogundijo et al. [36]. The findings of this study suggest that inorganic amendments increase the efficiency of organic fertilizers and vice versa [2,28] through positive interactions with soil nitrogen mineralization [37]. 

### 3.2. The Effects of Organic–Inorganic Amendments on Plant Growth

The effects of organic–inorganic soil amendments on spinach growth and biomass yield were evaluated in two contrasting soil types for eight weeks. The treatment combination of 2.5 g of dried algae and 0.6 g of N per kilogram of soil (50%DA50NF) consistently outperformed all other organic–inorganic amendments in both soil types. This included significantly (*p* < 0.05) higher values of plant height (Figure 6), leaf length (Figure 7), number of leaves (Figure 8), and biomass yield (Figure 9). Generally, loam soil produced better plant growth parameters and biomass yield compared with sandy loam soil (Figure 5, Figure 6, Figure 7, Figure 8 and Figure 9). Figure 5 is a visual demonstration of the differences in the vegetative growth of spinach during the third week after planting. The spinach with a higher biomass on the right was planted in loam soil and the one with a lower biomass on the left was planted in sandy loam soil (Figure 5). The observed and measured higher biomass yield in the loam soil is mainly attributed to the better soil fertility compared with the sandy loam soil (Table 3). For example, the total nitrogen and potassium contents were approximately three times higher in the loam soil compared with the sandy loam soil. In addition, the total carbon and phosphorus contents were approximately four times and six times higher in the loam soil, respectively, than in the sandy loam soil (Table 3). Moreover, the cation exchange capacity (CEC) of the loam soil was about four times higher in the loam soil, with approximately double the amount of calcium and magnesium compared with the sandy loam soil (Table 3). The higher cation exchange capacity of the loam soil could be attributed to the higher clay content, which was more than two times higher compared with that of the sandy loam soil. The high negative charge of clay surfaces and organic matter improves the CEC, which is important for retaining and making nutrients available to plants [9].

In addition to the differences in the chemical and physical properties between the two soil types, the three organic amendments significantly varied in their elemental compositions (Table 2). This may have influenced the overall soil fertility and crop productivity [34]; hence, the 50%DA50NF treatment combination produced a significantly (*p* < 0.05) higher biomass yield in both soil types compared with the ground grass and ground agri-mat treatments (Figure 9). Due to their low elemental composition, agri-mat residues were outperformed by the other two organic amendments in terms of nitrogen mineralization (Figure 1, Figure 2, Figure 3 and Figure 4) and spinach plant growth (Figure 6, Figure 7, Figure 8 and Figure 9). However, agri-mat can be favored under field conditions (especially as a mulching material) because it can produce a better biomass yield than grass material. Mgolozeli et al. [24] conducted field experiments to investigate the effects of agri-mat and grass mulch on spinach biomass yield in two different locations (one with the sandy loam soil type and another with the loam soil type) for two consecutive winter seasons. The findings from the study indicated that full agri-mat (100%AG) mulch treatment produced a 33% higher spinach biomass yield than the full grass mulch during the first season in the loam soil, with no percentage differences during the second season. For the sandy loam soil, the authors reported that agri-mat produced spinach biomass yields that were 20% and 32% higher during the first and second seasons, respectively, compared with the grass mulch cover [24]. Agri-mats are innovative pro-smallholder farmer permanent mulches that promote better soil quality and food security under conservation agriculture [23]. The current study corroborates the findings obtained by Mgolozeli et al. [22], who concluded that the incorporation of algae into agri-mat mulching technology could improve the functionality and value of agri-mats for agricultural purposes. This is based on the higher elemental composition (Table 2) and better nitrogen mineralization of algae residues observed in the current study compared with the other selected organic residues (Figure 1, Figure 2, Figure 3 and Figure 4). 

Agri-mats were initially designed, tested, and proven to be effective mulching materials that conserve soil moisture and prevent soil loss through erosion [38,39]. Further research and innovations in agri-mat mulching technology have indicated that agri-mats’ functionality, durability, and agricultural value can be improved by incorporating agrochemicals (chemical fertilizers, herbicides, and pesticides) during the fabrication process [22,39]. Therefore, agri-mats can suppress weed growth and reduce pest and insect infestation while combating soil erosion and conserving soil moisture [22,39]. Moreover, the incorporation of agrochemicals and different organic residues that vary in their C–N ratios (i.e., algae, manures, and grasses) may promote a sustainable environment and combat climate change [6,9,22]. This is because of the synergistic relationship between organic amendments and chemical fertilizers [2,28] through positive interactions with soil nitrogen mineralization and carbon sequestration [6,32,37]. Chemical fertilizers and other agrochemicals are easily lost through leaching in coarse-textured soil under field conditions [28]. Since the current results were obtained in a pot experiment with monitored irrigation, the net loss of N through leaching was minimized, even in the sandy loam soil. However, further incubation studies, glasshouse (pot) experiments, and field experiments are needed to determine the point at which all organic N is mineralized from different organic residues over longer periods. This will help to predict when to apply inorganic amendments to the soil under field conditions before planting. Moreover, this may also assist in quantifying the correct amounts of agrochemicals and appropriate organic amendment quantities during the agri-mat mulch fabrication process. 

## 4. Materials and Methods

### 4.1. Incubation Study

A 2-month incubation study was conducted to investigate the nitrogen mineralization of organic–inorganic amendments and organic amendments alone in two contrasting soil types in a laboratory at the Agricultural Research Council of the Natural Resources and Engineering campus in Pretoria, Gauteng, South Africa. The current study adopted and modified the methods described by Abbasi and Khaliq [8] and Shahbaz et al. [34]. Briefly, soil samples were collected from two different agroecological zones (Durban, which represented a humid region, and Pretoria, which represented a semi-arid region) in November 2018 at a depth of 0–0.30 m using a spade. The detailed textural analyses and physicochemical properties of the soils from each site are shown in Table 1 and Table 3, respectively. The soils were air-dried by spreading the samples on the floor of a well-ventilated room for a week to minimize microbial and chemical changes [11,40] and passed through a 2 mm sieve. A 100 g sample of each soil was transferred into 250 cm^3^ transparent plastic containers with perforated lids (to allow gaseous exchange) and treated with the required quantities of dry algae (DA), ground agri-mat (GAM), and ground grass (GG) and combined with lime–ammonium nitrate (28%) as a nitrogen fertilizer (NF). All the organic amendments (DA, GAM, and GG) were ground, oven-dried at 70 °C overnight, and passed through a 1 mm sieve [8]. Table 2 shows the chemical compositions of the three organic residues. The lime–ammonium nitrate (LAN (28%)) was produced by Sasol in Secunda, South Africa. The scientific name of the grass used was *Megathyrus maximus*, commonly known as Guinea grass. The grass was collected at the Agricultural Research Council, the Vegetable and Ornamental Plants Institute, at Roodeplaat, Pretoria. The ground agri-mat (GAM) was fabricated using forestry residues at Nagoya University (Nagoya, Japan) and shipped to South Africa. The algae residue used was green algae (*Chlorophyta*), which was collected from the Kingsburgh wastewater treatment plant in Durban after mechanical oil extraction. The algae were grown using an open-pond cultivation method with no interference from domestic waste processing in the plant and thus had no toxic heavy metals and organic pollutants. The extracted oil was then further processed for biofuel purposes. The algae residues were oven-dried at 70 °C overnight and freely given to any interested parties for agricultural purposes.

The application rates were calculated to provide an estimated amount of 150 kg N ha^−1^. This was obtained from a calculated bulk density of 1600 kg m^−3^ and 1500 kg m^−3^ for the sandy loam and loam soils, respectively, using a depth of 0.30 m. The 5 g of algae (with 6.8% N) was equivalent to applying 340 mg of nitrogen per kilogram of soil. Similarly, the ground grass (with 0.56% N) and ground agri-mat (0.25% N) were calculated accordingly to achieve 340 mg of nitrogen per kilogram of soil or 150 kg N ha^−1^. Thus, 2.5 g of dried algae, 61 g of ground grass, and 136 g of ground agri-mat were applied per kilogram of soil to achieve 150 kg N ha^−1^. Finally, the 50% treatment was calculated by dividing each organic material or compound fertilizer by two. Thus, the following treatments were achieved.

- T1 = control (no amendments);
- T2 = 5 g of dry algae per kg of soil (100%DA);
- T3 = 136 g of ground agri-mat per kg of soil (100%GAM);
- T4 = 61 g of ground grass per kg of soil (100%GG);
- T5 = 0.6 g of N using lime–ammonium nitrate (LAN) + 2.5 g of dry algae per kg of soil (50%DA50NF);
- T6 = 50%GAM50NF;
- T7 = 50%GG50NF.

Phosphorus and potassium were applied to the treatments at rates of 90 mg of 18% P_2_O_5_ kg^−1^ and 45 mg of 50% KCl kg^−1^ soil in the form of single superphosphate and muriate of potash (MOP), respectively. Both the 18% P_2_O_5_ and 50% KCl were produced by FERTASA, Pretoria, South Africa. After the treatment applications, the soil in all the units was irrigated with deionized water to an 80% field capacity initially, and every third day thereafter. Three samples were obtained per treatment at 0, 3, 7, 15, 30, 45, and 60 days for the N mineral (nitrate and ammonium) content determination. Therefore, the incubation study consisted of 7 treatments, 7 sampling dates (on days 0, 3, 7, 15, 30, 45, and 60), and 3 replications per sampling date, to make 147 experimental units (250 cm^3^ plastic containers with 100 g of soil) per sampling site at the beginning of the experiment. 

The experiment was laid out with a completely randomized design in an incubator set at 25 °C for 60 days (8 weeks). The soil water content was monitored throughout the incubation period according to Abbasi and Khaliq’s study [8] as follows: Every second day, the soil moisture content was checked and was adjusted back to an 80% field capacity by weighing the mass of each plastic container when the water loss was greater than 0.05 g. The deficit was added using deionized water to maintain the initial soil moisture content. During this process, precautionary measures were taken to avoid soil disturbance, through either stirring or shaking. Nitrate and ammonium contents were determined using a method described by Mulbry et al. [15]. Briefly, 10 g samples were extracted with 100 cm^3^ of 2 M KCL in a rotary shaker for 30 min. The extracts were filtered using a 0.45 µm membrane, and the pH of the filtrates was adjusted to 3–5 with H_2_SO_4_ as needed for preservation. The filtrates were stored frozen until further analysis. Ammonium and nitrate contents were determined calorimetrically by QuikChem^®^ 8500 series 2 flow injection analysis (Lachat Instruments, Milwaukee, WI, USA). The percentage increase in ammonium and nitrate ions was then calculated using Equation (1):

(1) $$\%\;\mathrm{difference}=\frac{\left|A-B\right|}{\frac{\left(A+B\right)}{2}}\times 100$$

where A is the value of ammonium or nitrate ions produced with organic–inorganic amendments (50%DA50NF, 50%GG50NF, and 50%GAM50NF) during peak nitrogen mineralization, while B is the value of ammonium or nitrate ions released with singular organic amendments (100%DA, 100%GG, and 100%GA).

### 4.2. Glasshouse Experiment

The glasshouse experiment was conducted for 2 months (8 weeks) at the Agricultural Research Council of the Vegetable and Ornamental Plants Institute in Roodeplaat, 35 km north-east of Central Pretoria, to determine the effects of organic–inorganic amendments on spinach growth and biomass yield. Soil was collected from each site at the same time as the incubation study and transferred into plastic pots. The length of the glasshouse experiment was informed by the results obtained in the incubation study as well as the test crop (spinach grown in pots). The pots had a height of 25 cm and a top diameter and bottom diameter of 20 cm and 15 cm, respectively. Each pot was treated with the required quantities of dried algae (DA), ground agri-mat (GAM), ground grass (GG), and nitrogen fertilizer (NF) using the following treatments for each soil type:

- T1 = control;
- T2 = 2.5 g of dry algae (DA) per kg of soil + 0.6 g of N per kg of soil using LAN (50%DA50NF);
- T3 = 68 g of ground agri-mat (GAM) per kg of soil + 0.6 g of N per kg of soil using LAN (50%GAM50NF);
- T4 = 30.5 g of ground grass (GG) per kilogram of soil + 0.6 g of N per kg of soil using LAN (50%GG50NF);
- T5 = 1.2 g of N per kg of soil using LAN (100NF).

The five treatments shown above were selected based on the results observed in the incubation study, which indicated that the combination of organic and inorganic amendments may have led to the release of ammonium and nitrate. The singular application treatments of organic materials did not lead to mineralization after 2 months of incubation (Figure 1, Figure 2, Figure 3 and Figure 4); hence, the singular treatments of organic amendments were omitted for the 2-month glasshouse experiment. Each treatment was replicated three times in a complete randomized design. Therefore, 15 pots per site (soil type) were established to make a total of 30 experimental units (pots). Table 2 shows the initial characterization of the three organic materials used.

Spinach (*Spinacia oleracea* L.) (cultivar: Fordhook Giant) was used instead of maize as the test crop for the glasshouse experiment due to the limited space for root growth in pot experiments. Three spinach seeds were planted per pot, all the pots were irrigated immediately after planting, and soil moisture was monitored afterward. The pots were weighed to determine the deficit, and distilled water was used to irrigate the soil to 80% of the field capacity every third day. In each case, the seedlings were thinned to one seedling per pot two weeks after sowing. Weeds were manually removed every other week, while pesticides were not applied due to no signs of pests or diseases throughout the experiment. The temperature in the glasshouse was regulated between 28 and 25 °C during the day and night, respectively. Growth parameters (plant height, leaf length, and number of leaves) were measured and recorded every two weeks until harvest in the eighth week. 

### 4.3. Statistical Analysis

A two-way analysis of variance (ANOVA) was carried out to test the effects of spinach development and yield parameters using the JMP 14.0 statistical software (SAS Institute, Inc., Cary, NC, USA). The mean comparison was performed using the least-significant difference test (LSD) at α = 0.05. 

## 5. Conclusions

The results from the incubation study indicate that at the peak of nitrogen mineralization, the amount of ammonium that was released from organic–inorganic amendments in the sandy loam soil was 178% to 195% higher than that released from the singular organic amendments. For nitrate ions, the amounts that were released ranged from 159% to 189% at the peak of N mineralization. Meanwhile, the amount of ammonium and nitrate ions released in the loam soil ranged from 157 to 186% and 153 to 167%, respectively. The injection of agrochemicals and the addition of grass and algal biomass into agri-mats during their fabrication process may improve their C–N ratio and nitrogen mineralization rate. This practice will help reduce environmental pollution through the efficient use of agrochemicals. Furthermore, it may help to reduce the impact of climate change through improved carbon sequestration in the soil. The glasshouse experiment showed that the 50%DA50NF treatment increased the spinach yield by 20.6% in sandy soil and 36.5% in loam soil compared with the second-best treatment (100NF). This suggests that algae-injected agri-mats containing about half the recommended amount of chemical nitrogen fertilizer can partially substitute the full recommended amount of chemical nitrogen fertilizer without affecting the yield and quality of spinach. However, field trials are needed to corroborate these findings, as the current experiment was conducted in a glasshouse using pot experiments.

## Figures and Tables

**Table 1:** Soil textural analyses of the sandy loam and loam soils.

	Sand					Silt		Clay
Sampling Site	Very Coarse (mm) 2–1	Coarse (mm) 1–0.5	Medium (mm) 0.5–0.25	Fine (mm) 0.25–0.1	Very Fine (mm) 0.1–0.05	Coarse (mm) 0.05–0.02	Fine (mm) 0.02–0.002	Clay (mm) <0.002
Sandy loam soil	4.00%	2.00%	17.20%	37.50%	15.60%	5.60%	8.70%	9.40%
Loam soil	3.90%	3.00%	9.70%	10.20%	11.00%	12.00%	25.90%	24.30%

**Table 2:** Elemental compositions of dry algae, ground agri-mat, and ground grass.

**Treatment**	**Tot C (%)**	**Tot N (%)**	**C–N Ratio**	**Ca (mg kg^−1^)**	**Mg (mg kg^−1^)**	**P (mg kg^−1^)**	**K (mg kg^−1^)**
Algae	45.00	6.80	6.6	14,000.19	8730.00	1037.00	5000.66
Agri-mat	46.00	0.25	184.0	2000.73	228.00	309.00	1000.58
Grass	42.40	0.56	75.7	1000.92	1920.70	687.00	1000.87
**Treatment**	**Fe (mg kg^−1^)**	**Al (mg kg^−1^)**	**Mn (mg kg^−1^)**	**Zn (mg kg^−1^)**	**B (mg kg^−1^)**	**Na (mg kg^−1^)**	
Algae	6270.00	1604.00	867.00	483.00	56.50	103.00	
Agri-mat	830.00	458.00	30.30	234.00	5.10	116.00	
Grass	8695.00	3635.00	240.10	54.60	5.86	277.00	

**Table 3:** Physicochemical properties of loam and sandy loam soils sampled at a top 30 cm depth.

Soil Properties	Pretoria	Durban
Physical characterization		
Textural class	Sandy loam soil	Loam soil
Bulk density (mg kg^−1^)	1530	1400
Chemical characterization		
pH in H_2_O (1:2.5)	6.20	6.50
Available P (mg kg^−1^)	3.11	19.97
Total N (%)	0.064	0.20
Total C (%)	0.59	2.30
C–N ratio	9.22	11.50
Exchangeable bases		
Ca (meq 100 g^−1^)	220.00	436.00
Mg (meq 100 g^−1^)	156.70	301.00
K (meq 100 g^−1^)	73.40	212.70
Na (meq 100 g^−1^)	74.00	84.40
CEC (meq 100 g^−1^)	12.00	46.00

## Data Availability

The data that support the findings of this study are available upon request from the corresponding author. They are not publicly available due to privacy restrictions.
